# Oroxylin A modulates mitochondrial function and apoptosis in human colon cancer cells by inducing mitochondrial translocation of wild-type p53

**DOI:** 10.18632/oncotarget.7927

**Published:** 2016-03-05

**Authors:** Chen Qiao, Na Lu, Yuxin Zhou, Ting Ni, Yuanyuan Dai, Zhiyu Li, Qinglong Guo, Libin Wei

**Affiliations:** ^1^ State Key Laboratory of Natural Medicines, Jiangsu Key Laboratory of Carcinogenesis and Intervention, China Pharmaceutical University, Nanjing 210009, People's Republic of China; ^2^ Department of Medicinal Chemistry, China Pharmaceutical University, Nanjing 210009, People's Republic of China

**Keywords:** p53, translocation, oroxylin A, oxidative stress

## Abstract

Oroxylin A is a flavonoid extracted from the root of Scutellaria baicalensis Georgi. We previously demonstrated that oroxylin A induced apoptosis in human colon cancer cells via the mitochondrial pathway. In the present study, we investigated the underlying mechanisms responsible for the mitochondrial apoptotic pathway triggered by oroxylin A. p53 regulates mitochondrial survival, mitochondrial DNA integrity, and protection from oxidative stress. We determined that oroxylin A induces p53 mitochondrial translocation and inhibits SOD2 activity. Additionally, our studies demonstrate that oroxylin A promotes the formation and mitochondrial translocation of the p53-Recql4 complex in HCT-116 cells. Finally, we showed that oroxylin A triggers cytosolic p53 activation, thereby promoting apoptosis. Mitochondrial translocation of p53 was also validated *in vivo*. Thus, oroxylin A induces mitochondrial translocation of p53 and leads to mitochondrial dysfunction in human colon cancer cells.

## INTRODUCTION

P53 is one of the most well-characterized proteins in cancer cell biology and is widely recognized as a tumor suppressor [1]. The functions of p53 include regulation of substrate metabolism, DNA repair, autophagy, cell senescence, angiogenesis, and oxidative stress [2]. Although most p53-dependent cellular processes are nuclear-based [3, 4], mitochondrial translocation of p53 has recently been detected raising the possibility of organelle-based effects [5, 6]. Mitochondrial translocation of p53 can suppress manganese superoxide dismutase (MnSOD), an antioxidant defense enzyme, and induce intracellular accumulation of reactive oxygen species (ROS) [7]. P53 can also activate several mitochondrial apoptosis-related proteinsF such as Bcl-xL to promote caspase activation and cytochrome C release [8].

The Recql4 protein is essential for p53 mitochondrial translocation and recruits p53 to the mitochondria in response to stress [9, 10]. Localization of p53 to the mitochondria via a mitochondrial localization signal can induce mitochondrial DNA replication and contribute to changes in the mitochondrial physiological status [9]. Oroxylin A (C_16_H_12_O_5_) is a flavonoid isolated from the root of traditional herbal medicine Scutellaria baicalensis Georgi. Recently, oroxylin A has been shown to have anticancer activities *in vitro* and *in vivo* through multiple mechanisms including cell cycle arrest, inhibition of metastasis, and apoptosis [11–13]. In this study, we provide evidence that oroxylin A facilitates mitochondrial translocation of p53 thereby inducing the mitochondrial apoptotic pathway.

## RESULTS

### Translocation of p53 to the mitochondria inhibits SOD2 activity and promotes ROS generation in wt-p53 cancer cells

**Figure 1:**
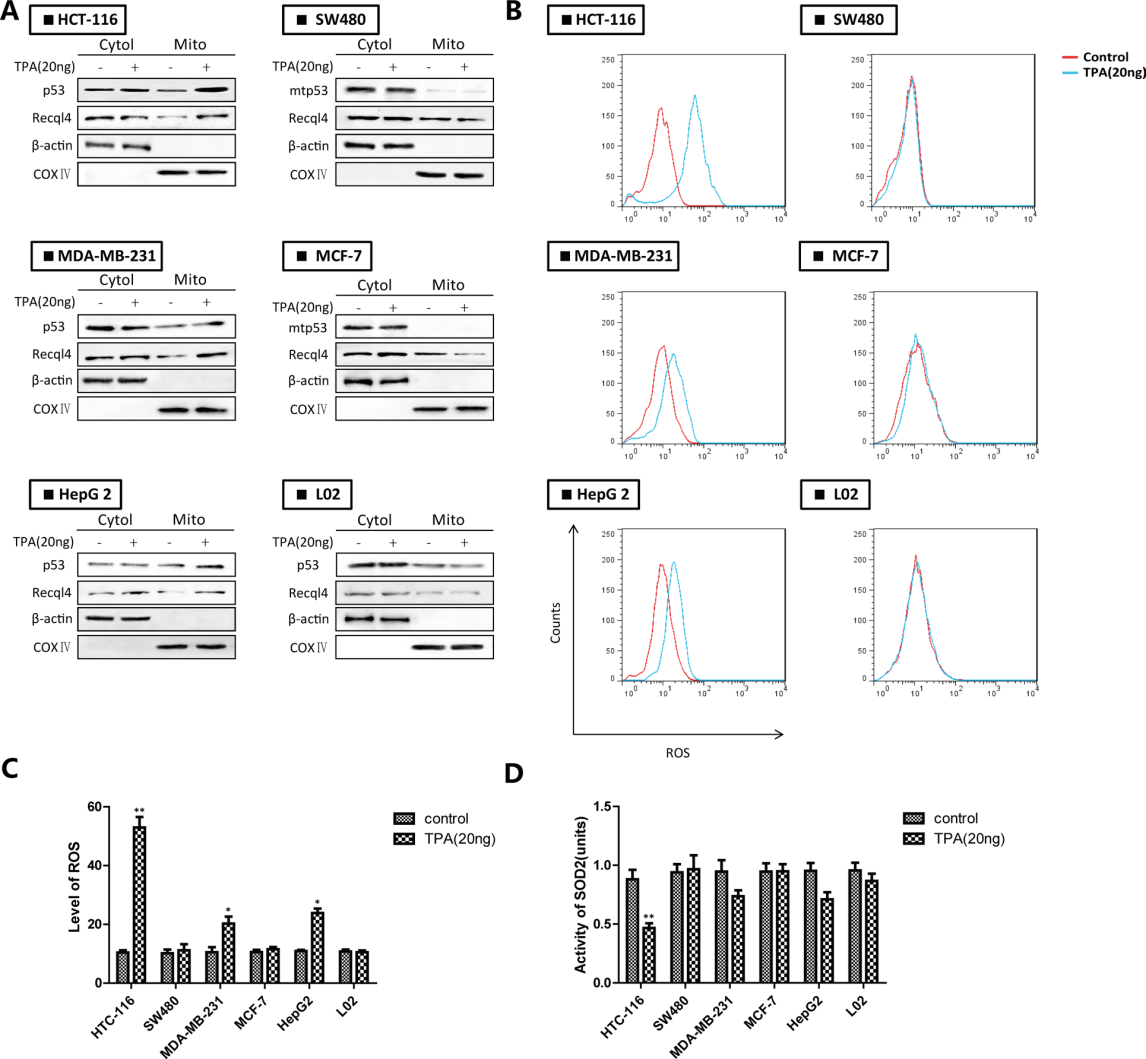
The accumulation of p53 in mitochondria leads to inhibition of SOD2 and increased ROS generation in wt-p53 cancer cells (**A**) Western blotting analysis of mitochondrial p53. (**B**) ROS production was monitored using 10 μM DCFH-DA and detected by flow cytometry. (**C**) Quantification of ROS levels. (**D**) Evaluation of SOD2 activity. Bar, SD. *P < 0.05 or **P < 0.01 versus the untreated control.

Wt-p53 rapidly translocated to mitochondria in response to apoptosis-induced stress signals (particularly ROS generation). To confirm wt-p53 translocation to the mitochondria in various cancer cells, we evaluated the subcellular distribution of p53 and ROS levels in wt-p53 cancer cells (e.g., HCT-116, MDA-MB-231, and HepG 2 cells), mut-p53 cancer cells (e.g., SW480, MCF-7), and normal cells (e.g., L02 cells) after TPA treatment [14]. After treatment of the cells with TPA for 24 h, p53 translocated to the mitochondria in the wt-p53 cancer cells but not in mut-p53 cancer cells or normal cells (Figure 1A). The ROS level in wt-p53 cancer cells showed a corresponding increase and SOD2 activity decreased (Figure 1B–1D). These results demonstrated that wt-p53 translocation contributed to inhibition of mitochondrial SOD2 activity and increased ROS levels in wt-p53 cancer cells.

### Oroxylin A induces apoptosis by promoting mitochondrial translocation of wt-p53 in HCT-116 cells

**Figure 2:**
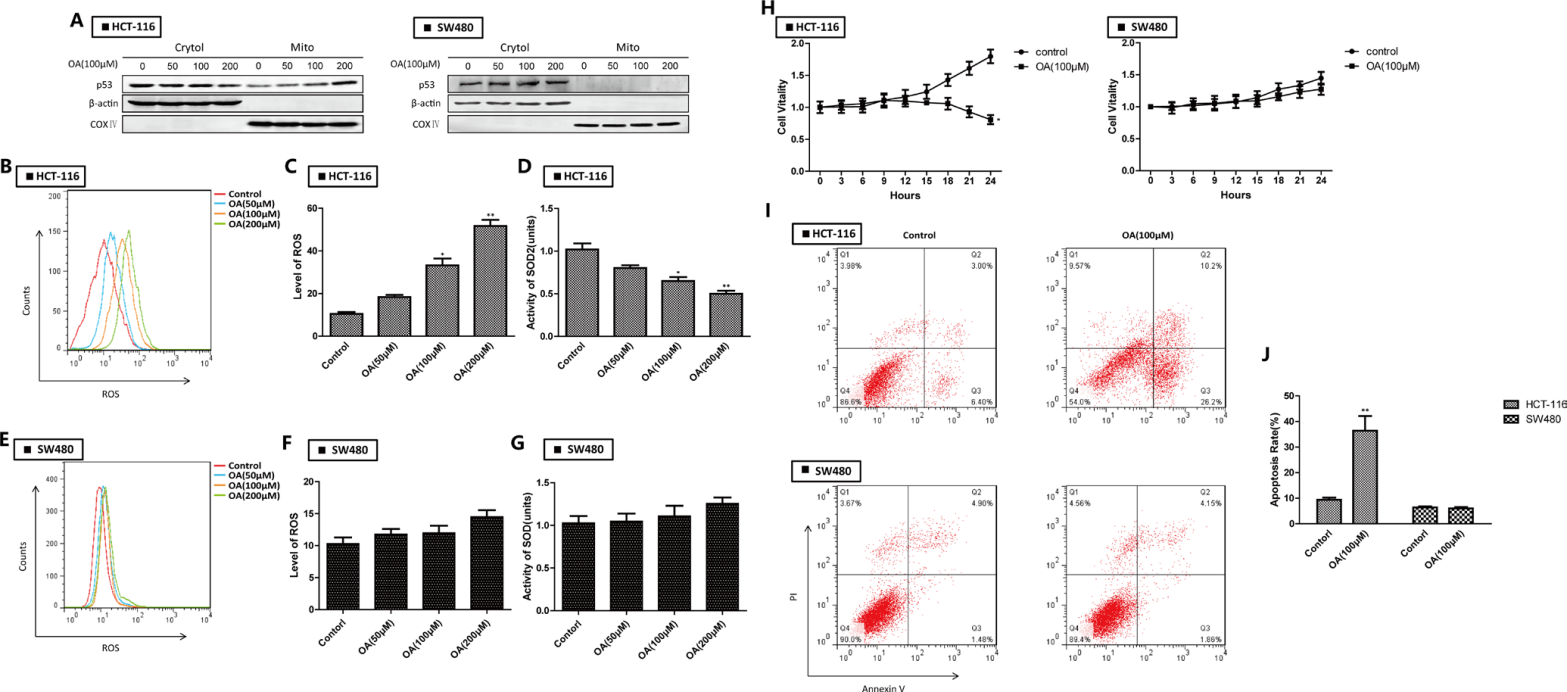
Oroxylin A induces apoptosis through p53 mitochondrial translocation in HCT-116 cells (**A**) Western blotting analysis of p53 accumulation in mitochondria after oroxylin A treatment (50 μM, 100 μM, and 200 μM) for 24 h. (**B**–**C**) ROS production was monitored using 10 μM DCFH-DA and then detected by flow cytometry after treatment of HCT-116 cells with 100 μM oroxylin A for 24 h. ROS levels were then quantified. Bar, SD. *P < 0.05 or **P < 0.01 versus the untreated control. (**D**) The activity of SOD2 was evaluated as described above. (**E**–**F**) ROS production was monitored in SW480 after treatment of with 100 μM oroxylin A for 24 h cells using 10 μM DCFH-DA and then detected by flow cytometry. ROS levels were then quantified. Bar, SD. *P < 0.05 or **P < 0.01 versus the untreated control. (**G**) The activity of SOD2 was evaluated as described above. (**H**) HCT-116 and SW480 cells were seeded in 96-well plates, incubated overnight, and treated with 100 μM oroxylin A. The results of these experiments are shown as the mean ± SD. (**I**–**J**) After treatment with 100 μM oroxylin A for 24 h, cells were stained with Annexin V and PI, and apoptotic cells detected by flow cytometry. Bar, SD. *P < 0.05 or **P < 0.01 versus the untreated control.

We previously demonstrated that oroxylin A had therapeutic potential in human colon cancer cells via a ROS-related mitochondrial pathway [13] in which p53 was stabilized and glycolysis inhibited [15]. Here, we determined that oroxylin A treatment increased the levels of mitochondrial p53 in HCT-116 cells in a dosage-dependent manner. In contrast, mitochondrial translocation of p53 was not observed in mut-p53 SW480 cells (Figure 2A). Oroxylin A increased ROS levels and decreased SOD2 activity (Figure 2B–2D) in HCT-116 but not in SW480 cells (Figure 2E–2G). The growth of HCT-116 cells was inhibited by oroxylin A while the growth of SW480 cells did not change significantly over time (Figure 2H–2J). These results indicated that oroxylin A promoted mitochondrial accumulation of wt-p53 resulting in inhibition of mitochondrial SOD2 activity, increased ROS levels, and induction of apoptosis.

### Oroxylin A-induced inhibition of SOD2 activity and increased ROS were mediated by wt-p53 mitochondrial translocation in HCT-116 cells

**Figure 3:**
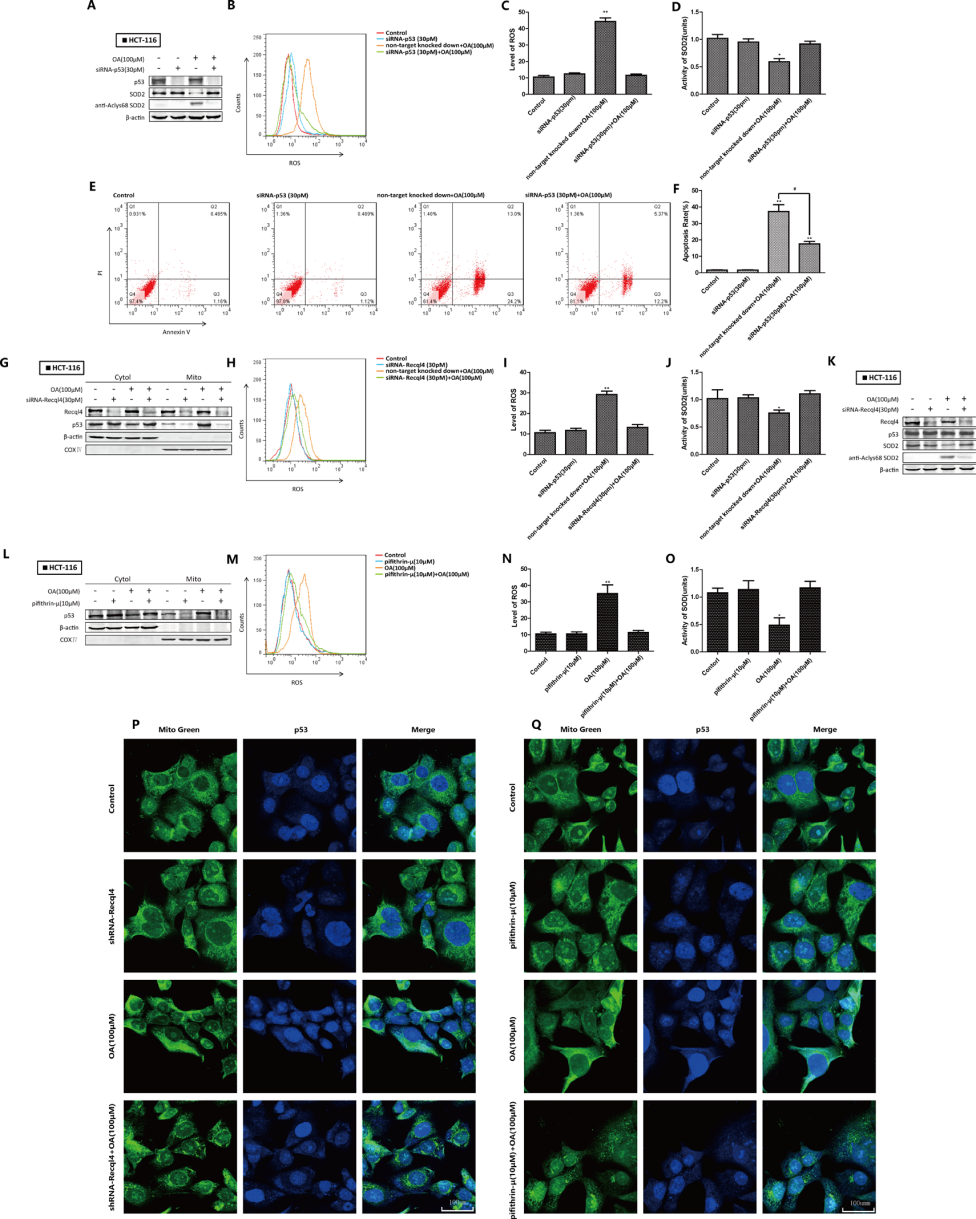
Oroxylin A-induced SOD2 activity inhibition and increased ROS were mediated by wt-p53 mitochondrial translocation in HCT-116 cells (**A**) Western blot detection of SOD2 expression in p53 siRNA-transfected HCT-116 cells after treatment with 100 μM oroxylin A for 24 h. (**B**–**C**) Quantification of ROS production in p53 siRNA-transfected HCT-116 cells. (**D**) SOD2 activity in p53 siRNA-transfected HCT-116 cells. (**E**–**F**) Quantification of p53 siRNA-transfected HCT-116 apoptotic cells. (**G**) The subcellular distribution of p53 in Recql4 siRNA-transfected HCT-116 cells as detected by Western blot after treatment with 100 μM oroxylin A for 24 h. (**H**–**I**) Quantification of ROS production in Recql4 siRNA-transfected HCT-116 cells. (**J**) SO2 activity in Recql4 siRNA-transfected HCT-116 cells. (**K**) Western blot analysis of p53 expression in Recql4 siRNA-transfected HCT-116 cells after treatment with 100 μM oroxylin A for 24 h. (**L**) HCT-116 cells were incubated with the indicated concentrations of DMSO, pifithrin-μ (10 μM), oroxylin A (100 μM), and pifithrin-μ (10 μM) + oroxylin A (100 μM) for 24 h, and the subcellular distribution of p53 analyzed by Western blotting. (**M**–**N**) ROS production in p53 pifithrin-μ (10 μM)-treated HCT-116 cells was monitored as described above and ROS levels quantified. (**O**) SOD2 activity in pifithrin-μ (10 μM)-treated HCT-116 cells was evaluated as described above. (**P**–**Q**) HCT-116 cells were incubated with the indicated concentrations of DMSO, shRNA/pifithrin-μ (10 μM), oroxylin A (100 μM), and shRNA/pifithrin-μ (10 μM) + oroxylin A (100 μM) for 24 h. Confocal images of the cells show the fluorescence of p53 in blue, Mito in green, and the merged images in Column 3. Bar, SD. *P < 0.05 or **P < 0.01 versus the untreated control.

We next investigated the importance of p53 in the oroxylin A-induced effects by silencing p53 in HCT116 cells. The level of AcLys68 SOD2 reflected the activation status. Knockdown of p53 attenuated the inhibition of SOD2 activity by oroxylin A (Figure 3A–3F). The oroxylin A-induced increases in ROS levels and apoptosis were simultaneously attenuated. Recql4 is essential for p53 mitochondrial translocation [9]. Following Recql4 knockdown, p53 could not localize to mitochondria. Oroxylin-induced inhibition of SOD2 activity and increased ROS were also weakened (Figure 3G–3J). Total p53 levels did not significantly change (Figure 3K). We also treated HCT-116 cells with pifithrin-μ, an inhibitor of p53 mitochondrial translocation, and found that the effects of oroxylin A were attenuated (Figure 3L–3O). Confocal imaging revealed that deletion of both Recql4 and pifithrin-μ inhibited oroxylin A-induced p53 mitochondrial translocation (Figure 3P–3Q). These results suggested that wt-p53 mitochondrial translocation had a key role in oroxylin A-induced inhibition of SOD2 activity and apoptosis in HCT-116 cells.

### P53 mutation inhibits oroxylin A-induced mitochondrial translocation

**Figure 4:**
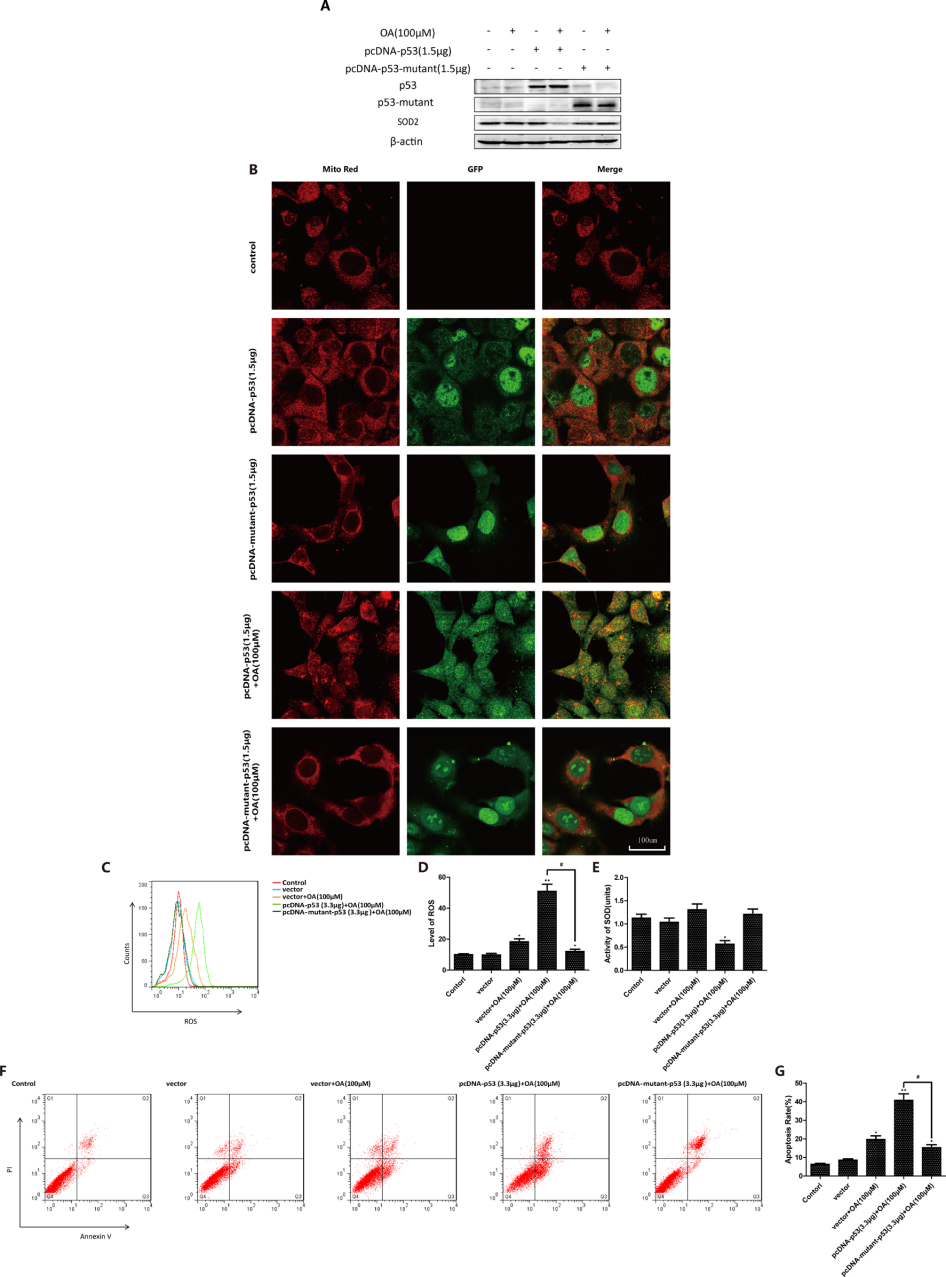
P53 mutation inhibits oroxylin A-induced mitochondrial translocation (**A**) P53-null HCT-116 cells were incubated with the indicated concentrations of DMSO, pcDNA-p53 (1.5 μg), oroxylin A (100 μM) + pcDNA-p53 (1.5 μg), pcDNA-mutant-p53 (1.5 μg), or oroxylin A (100 μM) + pcDNA-mutant-p53 for 24 h and SOD2 expressin detected by Western blotting. (**B**) Confocal images of the cells show the fluorescence of Mito in red, p53/mutant-p53 in green, and the merged image in Column 3. (**C**–**D**) ROS production was analyzed after transfection and oroxylin A treatment (100 μM) as described above. (**E**) SOD2 activity in p53/mutant-p53-transfected p53 null HCT-116 cells was evaluated as described above. (**F**–**G**) Apoptotic p53/mutant-p53 transfected p53-null HCT-116 cells were quantified as described above. Bar, SD. *P < 0.05 or **P < 0.01 versus the untreated control.

To further characterize the key roles of p53 in oroxylin A-induced mitochondrial translocation, we transfected p53-null HCT-116 cells with either wt-p53 or mut-p53. When cells were transfected with wt-p53, oroxylin A treatment induced release of p53 from the nucleus and mitochondrial translocation. However, when cells were transfected with mut-p53, p53 mitochondrial translocation was diminished (Figure 4A). In wt-p53-transfected HCT116 cells, oroxoylin A decreased the levels of SOD2, increased ROS generation, and induced apoptosis. In contrast, in mut-p53 transfected HCT-116 cells, these parameters were not significantly altered compared to vector-transfected control cells (Figure 4B–4F). The changes in SOD2 activity showed the opposite pattern (Figure 4E). Moreover, oroxylin A alone promoted mitochondrial translocation of p53 in Caco-2 (p53-null) cells transfected with wt-p53. Decreased SOD2 activity and increased ROS levels as well as apoptosis were observed (Supplementary Figure 1). These results indicated that mitochondrial translocation of p53 could be prevented when p53 was mutated. Oroxoylin A could only promote the mitochondrial translocation of wt-p53.

### Oroxylin A induces mitochondrial translocation of the p53-Recql4 complex and cytosolic activation of p53 in HCT-116 cells

**Figure 5:**
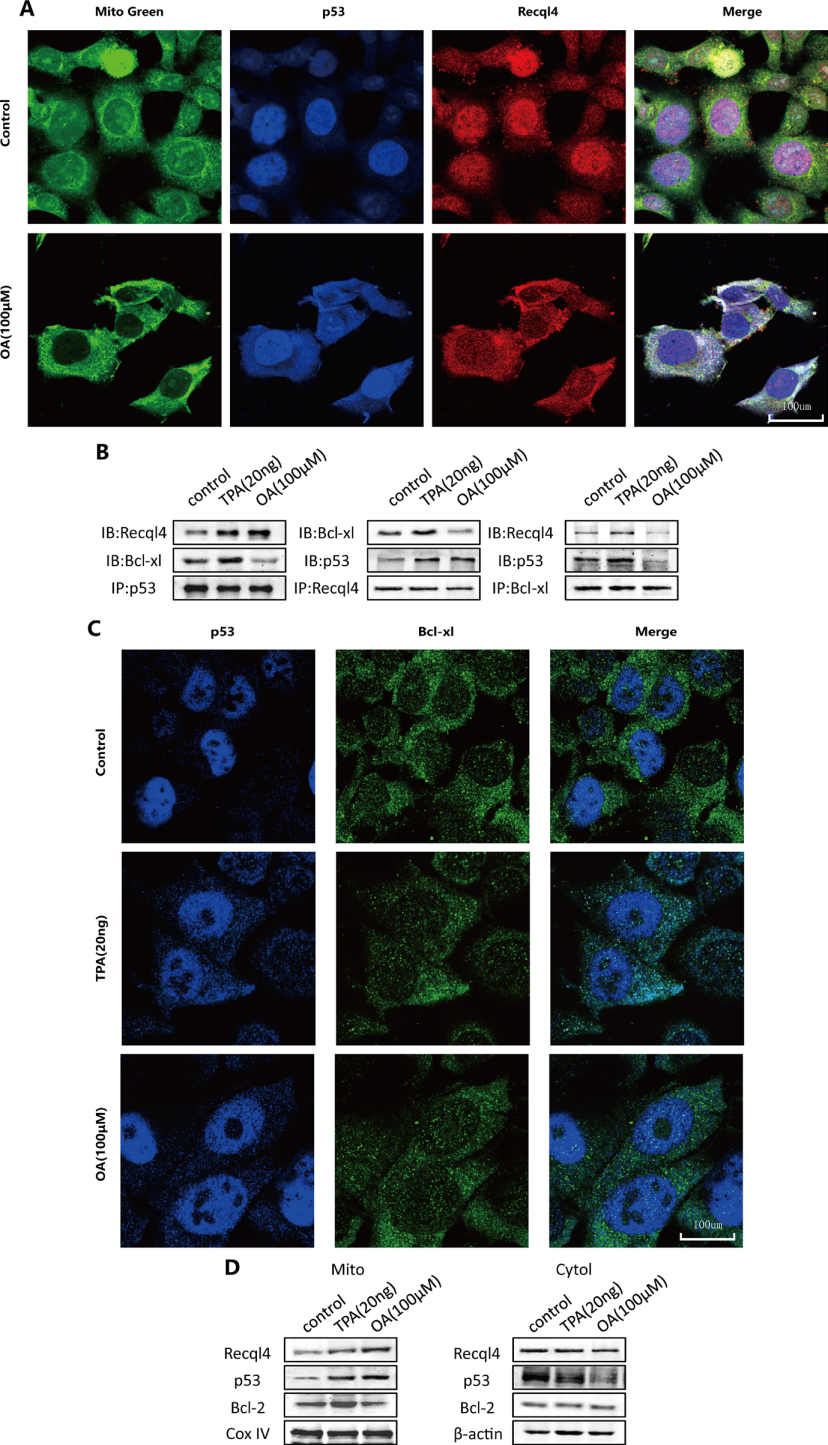
Oroxylin A induces cytosolic activation of p53 and recql4 mitochondrial translocation in HCT-116 cells (**A**) HCT-116 cells were incubated with the indicated concentrations of DMSO or oroxylin A (100 μM). Confocal images of the cells show the fluorescence of p53 in blue, Mito in green, Recql4 in red, and the merge in Column 4. (**B**) Antibodies to p53, Recql4, and Bcl-xL were used for the immunoprecipitation. Western blotting was used to detect complex formation between proteins. (**C**) HCT-116 cells were incubated with the indicated concentrations of DMSO, TPA (20 ng), and oroxylin A (100 μM). Confocal images of the cells show the fluorescence of p53 in blue, Bcl-xL in green, and the merged images in Column 3. (**D**) After the mitochondria were isolated from HCT-116 cells, Western blotting was performed to analyze Recql4, p53, and Bcl-2.

As shown in Figure 5A, mitochondrial translocation of the p53-Recql4 complex was observed in response to oroxylin A treatment. Previous studies have indicated that cytosolic p53 can be captured by Bcl-xL and sequestered into an inactive complex in unstressed cells [16]. Therefore, we investigated complex formation between p53 and Bcl-xL in HCT-116 cells using immunoprecipitation. The binding capacity of p53 and Bcl-xL increased in TPA-treated cells but decreased in oroxylin A-treated cells (Figure 5B–5C). Bcl-2 can interact with p53 and neutralize its proapoptotic effects on Bax and Bak, which oligomerize and form lipid pores in the outer mitochondrial membrane [16]. Mitochondrial Bcl-2 increased significantly in TPA-treated cells but decreased in oroxylin A-treated cells (Figure 5D). These results demonstrated that oroxylin A induced p53 cytosolic activation and mitochondrial translocation in the presence of Bcl-xL and Bcl-2.

### Oroxylin A induces mitochondrial translocation of p53 *in vivo*

**Figure 6:**
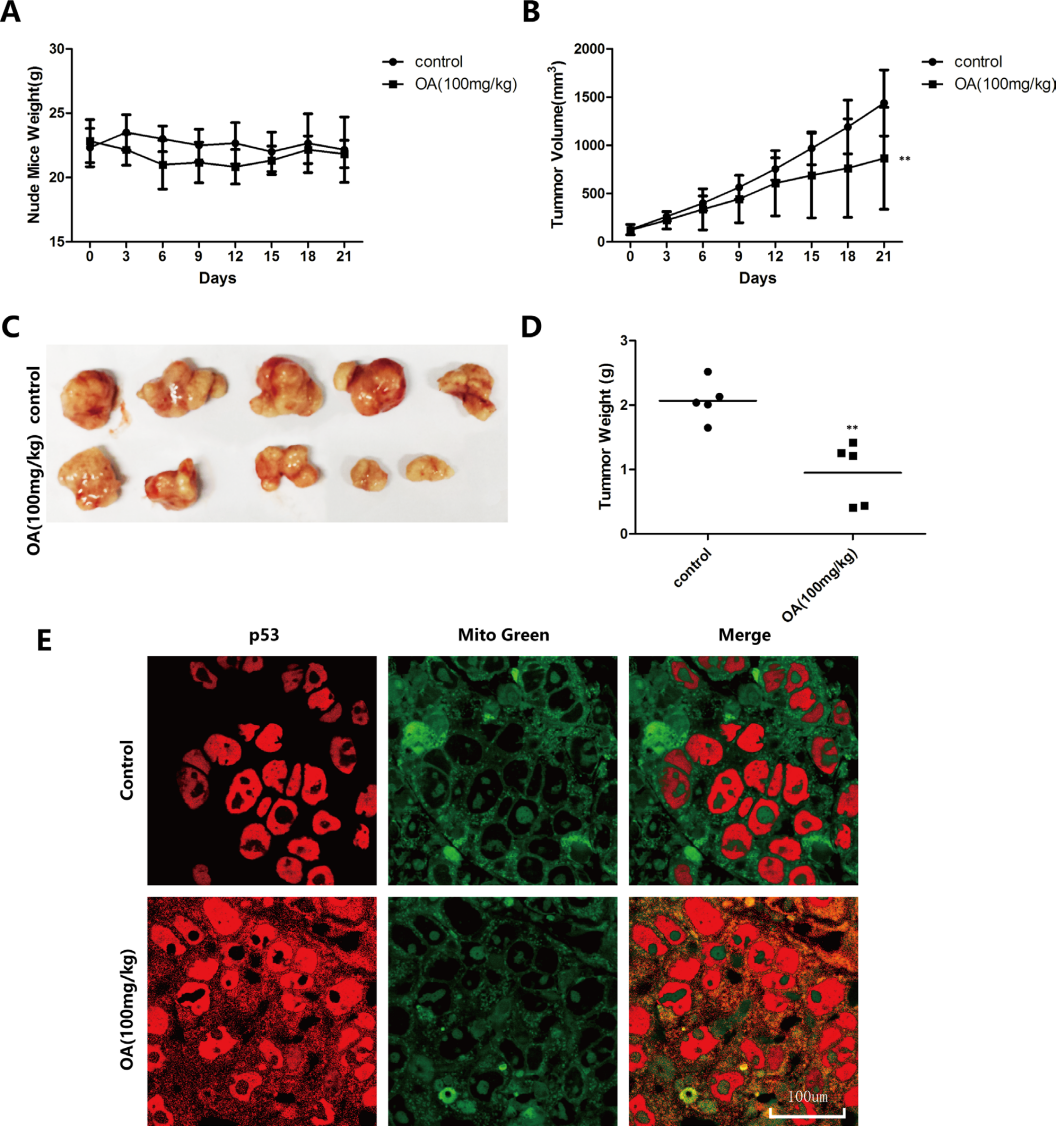
Oroxylin A induces Recql4-dependent mitochondrial translocation of p53 *in vivo* (**A**–**D**) HCT-116 tumors were transplanted into BALB/C nude mice and 100 mg/kg of oroxylin A administrated by intraperitoneal injection. (**E**) Confocal images of the cells show the fluorescence of p53 in red, Mito in green, and the merged images in Column 3.

To verify the oroxylin A-induced mitochondrial translocation of p53 *in vivo*, we used an HCT-116 transplanted tumor model established in BALB/C nude mice. We injected 100 mg/kg oroxylin A intraperitoneally and sections of human tumor tissue were obtained and analyzed by immunohistochemistry after 21 days. The results indicated that both the volumes and weights of the transplanted tumors decreased after oroxylin A treatment, while the weights of the nude mice did not change (Figure 6A–6D). The inhibitory rate of tumor growth in the oroxylin A-treated group reached 50.80%. Finally, wider distribution of p53 in the cytoplasm was observed by confocal microscopy compared to the control group (Figure 6E).

## DISCUSSION

Mitochondrial translocation of p53 has been reported to have a direct impact on mitochondrial function, similar to the nuclear effects of p53 [17–20]. We previously demonstrated that oroxylin A can induce the mitochondrial apoptotic pathway [21–24]. We hypothesized that mitochondrial translocation of p53 might be the internal mechanism by which oroxylin A triggers mitochondria-mediated apoptosis. The subcellular distribution in various tumor cells after TPA treatment in HCT-116 cells demonstrated the highest sensitivity. Therefore, we selected HCT-116 cells for further research. Western blotting of mitochondrial extracts revealed that wt-p53 translocated to the mitochondria in response to oroxylin A treatment in HCT-116 cells but not in SW480 cells in which p53 was mutated. HCT-116 cells were also more vulnerable than SW480 cells in which the mechanisms of ROS resistance might be suppressed by oroxylin A-induced mitochondrial translocation of wt-p53.

We achieved the same results in the absence of Recql4 and after treatment with an inhibitor of p53 mitochondrial translocation, indicating that p53 exerts its pro-apoptotic effects in the mitochondria rather than in the cytoplasm after oroxylin A treatment. These results were validated in p53-null HCT-116 cell model and p53-null Caco-2 cells (Supplementary Figure 1). Confocal microscopy revealed that wt-p53 was released from the nucleus. Thus, the first step of p53 translocation was confirmed in oroxylin A-treated cells. The other two essential elements for the process of p53 mitochondrial translocation are cytosolic transport and activation [25]. Dual fluorescence staining demonstrated p53 and Recql4 colocalization in mitochondria, indicating that transport was activated normally in response to oroxylin A. TPA has the ability to drive these two processes. However, it triggered invasion while oroxylin A induced apoptosis [26]. There might be some differences in p53 activation in oroxylin A-treated cells compared to TPA-treated cells. Cytosolic p53 activity can be captured and neutralized by cytosolic Bcl-xL through formation of an inactive complex [27]. After TPA treatment, complex formation between Bcl-xL-p53 and p53-Recq14 increased, indicating initiation of a protective mechanism. After oroxylin A treatment, binding of p53-Recq14 increased while Bcl-xL was liberated from the complex, allowing activated p53 to be transported. Finally, we verified oroxylin A-induced p53 translocation *in vivo*. As shown in Figure 5E, p53 exhibited a wider distribution in mitochondria.

In summary, our studies of HCT-116 colon cancer cells have shown that oroxylin A can induce the cytosolic activation and mitochondrial translocation of p53, which triggers the mitochondrial apoptotic pathway. We have elucidated the role of p53 mitochondrial translocation in oroxylin A-induced apoptosis, demonstrating that oroxylin A has the capacity to selectively inhibit tumor cells and lower toxicity. This could be applied to the development of anti-tumor drugs.

## MATERIALS AND METHODS

### Materials

Oroxylin A was isolated from the root of *Scutellaria baicalensis* Georgi and dissolved in dimethyl sulfoxide (DMSO) according to previously published protocols. Samples that contained ≥ 99% oroxylin A were used in all experiments. Oroxylin A was dissolved in DMSO to 200 mM and stored at −20°C. Before every experiment, a stock solution of oroxylin A was diluted in basal medium to various working concentrations. The 12-O-tetradecanoylphorbol 13-acetate (TPA) was purchased from Sigma-Aldrich (St. Louis, MO, USA) and dissolved in normal saline.

### Cell culture

HCT-116, SW480, Caco-2, MDA-MB-231, MCF-7, HepG 2, and L02 cells were purchased from the Cell Bank of Shanghai Institute of Biochemistry and Cell Biology, Chinese Academy of Sciences (Shanghai, China). HCT-116 cells were maintained in 90% McCoy's 5A medium (Sigma-Aldrich) supplemented with 10% heat-inactivated fetal bovine serum (FBS) (GIBCO, Invitrogen, Carlsbad, CA, USA), 100 U/mL penicillin G, and 100 μg/mL streptomycin. SW480, Caco-2, MDA-MB-231, and Hep G2 cells were maintained in 90% Dulbecco's Modified Eagle Medium supplemented with 10% heat-inactivated FBS (Sijiqing, Hangzhou, China). MCF-7 cells and L02 cells were maintained in 90% 1640 medium supplemented with 10% heat-inactivated FBS (Sijiqing, Hangzhou, China). The p53-null HCT116 cell line was provided by the Life Sciences Institute at the University of Michigan. All cell types described above were cultured at 37°C in a water-jacketed CO_2_ incubator (Thermo Fisher Scientific, Waltham, MA, USA) in a humidified atmosphere with 5% CO_2_.

### Animal model

Male athymic BALB/c nude mice (35–40 days old) with body weights ranging from 18–22 g were supplied by the Academy of Military Medical Sciences of the Chinese People's Liberation Army (Certificate No. SCXK-[Army] 2007–004). Animals were maintained at 22 ± 2°C with 55–65% humidity in stainless steel cages under controlled light (12 h light/day) and were fed with standard laboratory food and water. Animal care was provided in accordance with the Guide for the Care and Use of Laboratory Animals published by the United State National Institute of Health.

Twenty nude mice were inoculated subcutaneously with 1 × 10^7^ HCT-116 cells into the right axilla. After 12 days of growth, tumor size was measured using micrometer calipers. Mice that were inoculated with HCT-116 cells and had similar tumor volumes were randomly divided into the following two groups (five mice/group): saline control and oroxylin A (100 mg/kg intravenously every 2 days). Tumor size was measured every 3 days using micrometer calipers and tumor volume was calculated using the following formula: Tumor volume (mm^3^) = d^2^ × D/2, in which d and D were the shortest and the longest diameters, respectively. Mice were sacrificed on day 21 and tumor tissues analyzed by immunohistochemistry.

### Alamar blue assays

HCT-116 and CaCo-2 cells were seeded in 96-well plates, incubated overnight, and the treated with 100 μM oroxylin A. Alamar blue assays (Invitrogen) were then performed according to the manufacturer's instructions. Fluorescence was analyzed once an hour for 9 h at an excitation wavelength of 530–560 nm and an emission wavelength of 590 nm using a fluorospectrophotometer (Olympus, Japan). The Alamar blue assay incorporates a fluorometric/colorimetric growth indicator based on detection of metabolic activity. Specifically, the system incorporates an oxidation-reduction indicator that both fluoresces and changes color in response to chemical reduction of the growth medium that results from cell growth.

### Western blotting

Proteins were isolated using lysis buffer, incubated in SDS buffer, separated by SDS-PAGE, and transferred onto PVDF membranes. Antibodies to Bcl-2 (sc-7382), p53 (sc-6243), Recql4 (sc-366840), and SOD2 (sc-133134) were purchased from Santa Cruz Biotechnology (Santa Cruz, CA, USA). The antibody to Bcl-xL (2764p) was purchased from Cell Signaling Technology (Danvers, MA, USA). Antibodies to Cox IV (ab14744) and SOD2 acetylated at Lysine 68 (AcLys68) (ab137037) were purchased from Abcam (Hong Kong, China), and the antibody to β-actin (BM0627) was purchased from Boster Biotechnology (Wuhan, China).

### Annexin V/propidium iodide staining

Apoptotic cells were identified using Annexin V-FITC Apoptosis Detection Kits (KeyGen, Naijing, China) according to the manufacturer's instructions. In brief, 1 × 10^6^ cells were harvested, washed, and suspended in PBS. Cells were then resuspended in 500 μL Binding Buffer, and 5 μL AnnexinV-FITC and 5 μL propidium iodide were added. Apoptotic cell death was analyzed using FACSCalibur flow cytometry (Becton Dickinson, San Jose, CA, USA) immediately after double supravital staining.

### Measurement of intracellular ROS

Intracellular ROS was detected by DCFH-DA (Beyotime Institute of Biotechnology, Haimen, China). Cells were harvested from 6-well plates, washed with PBS, resuspended in serum-free medium, and then incubated with 10 μM DCFH-DA for 30 min at 37°C in the dark. The fluorescence intensity (488 nm excitation and 525 nm emission) was measured immediately using FACSCalibur flow cytometry (Becton Dickinson).

### SOD2 activity assays

SOD2 activity was assayed using the Cu/Zn-SOD and Mn-SOD Assay Kit (KeyGen) using the manufacturer's protocols. Briefly, cells were first collected and lysed, and then 3 mM potassium cyanide was added to the lysate to inhibit both Cu/Zn-SOD and extracellular SOD, which allowed detection of Mn-SOD activity. Samples were assayed in the absence of xanthine oxidase to generate a background sample. After the samples and SOD standards were prepared and added to a 96-well plate, the reaction was initiated by adding 20 μL of diluted xanthine oxidase to all wells. The plate was then incubated on a shaker for 30 min at room temperature. Optical density values were detected using a spectrophotometer (Thermo Fisher Scientific) at 450 nm.

SOD activity was quantified using the following equation obtained from the linear regression of the standard curve substituting the linearized rate (LR, LR = (A_blank1_-A_blank2_-A_sample_)/(A_blank1_-A_blank2_) × 100%) for each sample. One unit was defined as the amount of enzyme that exhibited 50% dismutation of the superoxide radical. Thus, SOD activity _sample_(U) = LR _sample_ /(1-LR _sample_) units.

### Transient transfection of plasmids and siRNA

The pcDNA3-p53-GFP and pcDNA3-p53-mutant-GFP plasmids were obtained from Addgene (Addgene 11770; Addgene 24859, Addgene Cambridge, MA, USA). The siRNAs for p53 and Recql4 were purchased from Santa Cruz Biotechnology. For transfection, HCT-116 cells were seeded in 6-well plates at 70% confluency, and then siRNA-p53 (30 pM)/siRNA-Recql4 (30 pM) were introduced into the cells using Lipofectamine 2000 (Invitrogen) according to the manufacturer's instructions. P53 null HCT-116 cells and Caco-2 cells were incubated with pcDNA-p53-GFP/pcDNA-p53-mutant-GFP and transfected using the same method. The cells were then exposed to oroxylin A or vehicle and harvested for further analysis.

### Stable transfection of shRNA

The Recql4 shRNA plasmid (TL309882) was obtained from Origene (Rockville, MA, USA). HEK293 cells were seeded at a density of 3 × 10^5^ cells in 2 mL into one well of a 6-well plate. The cells were then transfected with the Recql4 shRNA plasmid using Lipofectamine 2000 (Invitrogen) according to the manufacturer's recommendations Selection pressure was maintained by the presence of approximately 0.5–1.0 μg/mL puromycin. After the packaged cells reached approximately 70–80% confluency, the culture media was collected and passed through a 0.45 μm filter. The virus stock and 4 μg/mL polybrene in growth medium were then added directly to the target cells.

### Immunofluorescence

Cells were rinsed with PBS and fixed with 4% paraformaldehyde in phosphate buffer (pH 7.4) for 20 min at room temperature. After washing with PBST, the cells were blocked with 3% bovine serum albumin in PBST for 1 h, incubated with anti-p53 antibody/anti-Recql4 antibody (Santa Cruz Biotechnology) overnight at 4°C, and then washed with PBST. Tetramethylrhodamine-labeled anti-mouse/anti-rabbit IgG antibody (Rockland, Limerick, PA, USA) was then added and the cells incubated for 1 h. Mitochondria were identified by Mito Red (KeyGen) according to the manufacturer's instructions. Finally, the cells were rinsed with PBST and exposed to DAPI for 15 min to stain the nuclei. After washing with PBS, the cells were examined under a laser scanning confocal microscope FV10-ASW [Ver 2.1] (Olympus Corp, MPE FV1000) for co-localization analysis.

### Tissue immunofluorescence

Tissue sections (4 mm thick) were dewaxed and rehydrated through graded washes of alcohol in distilled water (100, 95, 85, 75%). The sections were then boiled in citrate buffer at 98°C for antigen retrieval and treated with 3% hydrogen peroxide to block endogenous peroxidase activity. Finally, the sections were incubated with a protein-blocking agent (kit 9710 MAIXIN, Maixin-Bio Co., Fuzhou, Fujian) prior to the application of the primary antibody. Slides were incubated with the anti-p53 antibody (Santa Cruz Biotechnology) overnight at 4°C and then washed with PBST. Mitochondria were identified using Mito Red (KeyGen) according to the manufacturer's protocol. After washing with PBS, tissues were examined under a laser scanning confocal microscope FV10-ASW [Ver 2.1] (Olympus Corp, MPE FV1000).

### Immunoprecipitation

Recaql4, Bcl-xL, and p53 were immunoprecipitated from the total protein using polyclonal antibodies crosslinked to protein G-agarose beads (Santa Cruz Biotechnology). The immune complexes were analyzed by Western blotting and probed with antibodies against Bcl-xL, p53 of interest.

### Preparation of mitochondria- and cytosol-enriched extracts

Following the 48 h incubation of the cells with oroxylin A, mitochondria/cytosolic fractionation was then performed using the Mitochondria/Cytosol Fractionation Kit (BioViso, VA, USA) according to the manufacturer's protocol.

### Statistical analysis

The data are presented as the mean ± standard deviation (SD) from triplicate parallel experiments unless otherwise indicated. Statistical analyses were performed using one-way ANOVA.

## SUPPLEMENTARY MATERIALS FIGURE


