# Supplementary material for: Oroxylin A modulates mitochondrial function and apoptosis in human colon cancer cells by inducing mitochondrial translocation of wild-type p53
Source: Oncotarget. 2016 Mar 5;7(13):17009–20. doi: 10.18632/oncotarget.7927 (PMC4941367; doi:10.18632/oncotarget.7927)
Supplement: Supplementary file 1 [file oncotarget-07-17009-s001.pdf]

Oroxylin A modulates mitochondrial function and apoptosis in human colon cancer cells by inducing mitochondrial translocation of wild-type p53

Supplementary Materials

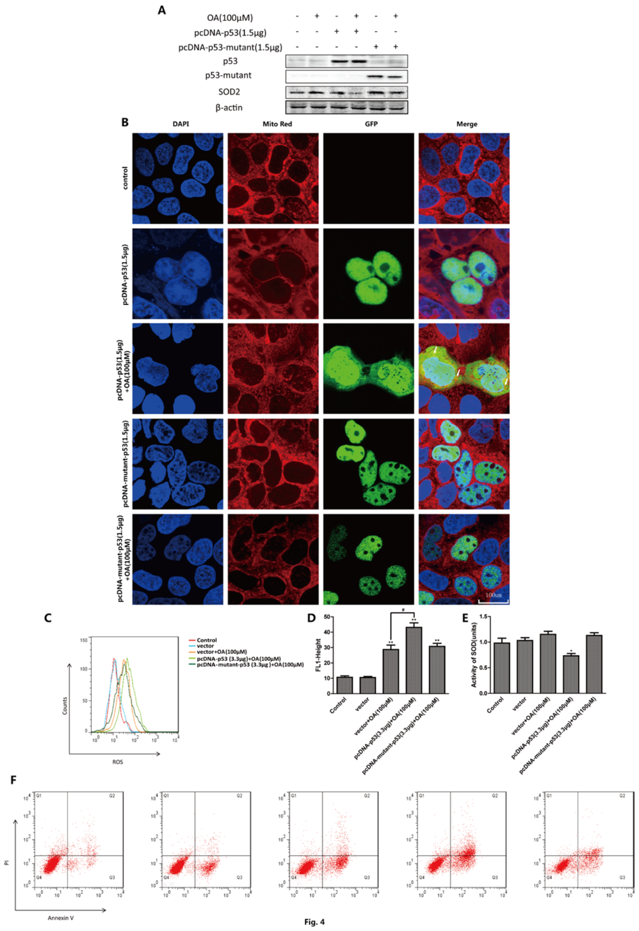

Supplementary Figure S1: (**A**) CaCo-2 cells were incubated with indicated concentration of DMSO, pcDNA-p53 (1.5 μg), oroxylin A(100 μM) + pcDNA-p53 (1.5 μg), pcDNA-mutant-p53 (1.5 μg), oroxylin A(100 μM) + pcDNA-mutant-p53 for 24 h and SOD2 expression were detected by Western blot. (**B**) Confocal images of cells show fluorescence of DAPI in blue, Mito in red, p53/mutant-p53 in green and are merged in Lane 4. (**C**) The ROS production was tested after transfection and oroxylin A (100 μM) as above(**D**) The ROS levels were quantified. (**E**) Activity of SOD2 in p53/mutant-p53 transfected CaCo-2 cells was evaluated as above. (**F**) Apoptosis cells in p53/mutant-p53 transfected CaCo-2 cells were quantitated as above. Bar, SD. *$P < 0.05$ or **$P < 0.01$ versus untreated control.
